# What students do schools allocate to a cognitive-behavioural intervention? Characteristics of adolescent participants in Northern Sweden

**DOI:** 10.3402/ijch.v74.29805

**Published:** 2015-11-03

**Authors:** Heléne Zetterström Dahlqvist, Evelina Landstedt, Katja Gillander Gådin

**Affiliations:** Department of Health Sciences, Mid Sweden University, Sundsvall, Sweden

**Keywords:** cognitive-behavioural interventions, school-based, selective interventions, depressive symptoms, naturalistic setting, psychosocial factors, sexual harassment

## Abstract

**Background:**

Adolescents are a vulnerable group when it comes to the risk of developing depression. Preventing the onset of depressive episodes in this group is therefore a major public health priority. In the last decades, school-based cognitive-behavioural interventions have been a common primary prevention approach. However, evidence on what girls actually are allocated to such interventions when no researchers are involved is scarce.

**Objective:**

To explore how a selective cognitive-behavioural program (Depression In Swedish Adolescents) developed to prevent depression in adolescents, was implemented in a naturalistic setting in schools in northern part of Sweden. The focus was on characteristics of participants allocated to the intervention.

**Design:**

Cross-sectional baseline data on depressive symptoms, school environment and socio-economic factors were collected in 2011 by means of questionnaires in schools in a municipality in the northern part of Sweden. Intervention participants were identified in a follow-up questionnaire in 2012. Students (n=288) included in the analyses were in the ages of 14–15.

**Results:**

Sixty-six girls and no boys were identified as intervention participants. They reported higher levels of depressive symptoms, lower personal relative affluence, more sexual harassment victimization and less peer support compared to female non-participants (n=222). Intervention participants were more likely to attend schools with a higher proportion of low parental education levels and a lower proportion of students graduating with a diploma.

**Conclusions:**

The developers of the intervention originally intended the program to be universal or selective, but it was implemented as targeted in these schools. It is important for school administrations to adhere to program fidelity when it comes to what students it is aimed for. Implications for effectivenss trials of cognitive-behavioural interventions in the school setting is discussed.

Between 1990 and 2010 mental health problems among adolescents have increased in Sweden but not in the other Nordic countries (1). Preventing the onset of depression in early age is of paramount importance to ensure a positive health development for young people. In the last decades, school-based cognitive-behavioural interventions have been a common primary prevention approach, but evidence of the effectiveness of these is inconsistent (2–4). These preventive intervention programs may be designated either for a general population (i.e. universal intervention), for groups with elevated risk (i.e. selective intervention), or for those who already have symptoms and/or a diagnosis (i.e. targeted intervention). Selective programs have been found to be more effective than universal ones (5). However, several interventions have been implemented as universal, for example, LARS&LISA (6) and the Penn Resiliency Program (7,8). Both of these programs have been shown to have effect on depressive symptoms outcomes in randomized controlled trials. LARS&LISA was reported to be effective at a 4-month follow-up (6), and the Penn Resiliency Program for adolescents contributed to reducing depressive symptoms as well as hopelessness (7,8).

A *targeted* intervention (subjects had established depressive symptoms) of the American Adolescent Coping with Depression Course (CWDA) was shown to be effective in preventing affective disorder at a 12-month follow up (9). In Sweden, a shortened and widely used version of the CWDA, called Depression In Swedish Adolescents (DISA), has been developed by Clarke and colleagues together with Centre for Public Health at Stockholm County Council (10). Modification and adjustment of the original manual and workbook have been made to mirror Swedish conditions and school curriculum (10). The Swedish developers consider the DISA program to be a *universal* preventive intervention for girls and boys, but also a *selected* program aimed for girls, because being female is a risk factor for depressive symptoms (11). The developers stress that the intervention is not a targeted one, that is, should not be considered as treatment for mood disorders (11). Notably, however, the effectiveness of DISA has only been scientifically evaluated in the Swedish context in 2 previous studies with discrepant results. One quasi-experimental study with no comparison group (12) implemented DISA as a universal intervention offering participation to both sexes (12). Participants were recruited by offering all students in grade 8 (14 years old) in a single school, located in an area with high mean income, to participate. At baseline, the mean and median scores on depressive symptoms were below the threshold for depressive symptoms, and the authors (12) report a significant reduction in depressive symptoms in girls at post-intervention and at 12-month follow-up. In boys there was a significant decrease in symptoms at post-intervention only. However, the lack of a comparison group in their study limits the conclusions that can be made regarding effectiveness. Furthermore, no possible confounders were controlled for (12). The other study was conducted by Treutiger and Lindberg (10) and included girls only making it a selective intervention. The investigators reported effect on depressive symptoms at a 3-month follow-up but not after 12 months. The mean score at baseline was below the threshold for depressive symptoms (10); however, the investigators also made separate analyses for high risk girls (intervention group, *x*=23.04; and a waiting list condition, *x*=23.65) at baseline and could not show effect at the 3-month follow-up or at the 12-month follow-up. This indicates that the DISA program may not have an effect on elevated depressive symptoms.

## Method development and aim of DISA

The DISA program has been adapted from cognitive therapy methods for adults and builds on Lewinsohn's and Clarke's (13) “The Coping with Depression Course” for adults and cognitive-behavioural treatment for depressed adolescents later developed by Lewinsohn and colleagues (14). The DISA program's main objective is to control irrational and negative thoughts. Also, it includes a conflict-resolution component wherein communication and problem solving skills are learned. Furthermore, the intervention focuses on encouraging girls to increase pleasant events in their lives (11).

DISA is manual-based and consists of 10 group sessions of 1½ hours each over 10 weeks, a total of 15 hours. These sessions can be delivered within school hours or as an extracurricular activity. The first session gives a general introduction of DISA. The following 2 sessions consist of an overview of depressive symptoms and the connection to stressful events. Sessions 4–6 focus on teaching cognitive skills to identify and cope with irrational or negative self-perceptions and thought patterns. The remaining sessions cover communication and problem solving skills. In addition, homework assignments are included as part of the training (11).

## DISA in the Nordic context

The DISA program is common in Sweden and has also been used in the Swedish-speaking part of Finland (15). The program is delivered in Swedish, and to our knowledge, there have been no translations to other languages. In contrast to researcher-led interventions, DISA is facilitated by school nurses, teachers and school counsellors or youth workers. It is recommended that schools implement the intervention to all students in grade 7 or 8. However, school resources may be limited, and hence, schools are able to implement DISA to only some of the students. If this is the case, students may be allocated to a DISA group either based on each school's assessments of whom they find appropriate for the intervention, for example, by offering the intervention to a particular group of students, or on a first come first served basis, via a general invitation to participate. In some cases, the choice of students reflects timetabling (personal communication, Eva-Mari Thomas, 3 September 2015).

Despite the lack of scientific evidence of its effectiveness in the Swedish context, the DISA method has been used in schools throughout Sweden. However, to our knowledge, there are no studies in Sweden or elsewhere of how schools implement school-based cognitive-behavioural programs with regard to characteristics of the students allocated to participate. This is of interest as DISA was developed as a selective (or universal) intervention and should not be delivered as a targeted intervention to students with elevated depressive symptoms (11).

## The DISA program as implemented by schools

Although randomized controlled trials are the preferred design in effect evaluations, it is important to investigate how schools actually implement these methods when no researchers are involved. Even though naturalistic designs often are associated with qualitative research (16), knowledge that can be gained through such designs is needed to extend the evidence regarding these cognitive-behavioural interventions (17). As demonstrated by Harnett and Dadds (18) and Spence and colleagues (19), research on how schools implement programs, regarding the quality of program delivery and facilitator competence is important. Also, in order to enhance the understanding of the environmental and individual circumstances under which mental health interventions are optimally effective, it is crucial to explore what characterizes such circumstances. As called for by Sutton (20), school environmental and socio-economic factors should be investigated as possible moderators or confounders of the effects of an intervention. To our knowledge, the current study is the first to examine how the DISA program is implemented in a naturalistic setting regarding characteristics of recruited students.

## Objective

The main objective was to investigate how DISA was implemented in schools regarding what features characterize students who were allocated to the intervention. Five specific research questions were outlined: (a) Were DISA participants represented by both sexes? (b) Were there differences of depressive symptoms between the implementation and comparison groups? (c) Did the implementation and comparison groups report different levels of peer and teacher support, school demands, sexual harassment and bullying victimization? (d) Were there differences in socio-economic status between the implementation and comparison groups? and (e) Were there socio-economic differences between schools in which the intervention was widely implemented compared to schools with few or no intervention participants?

## Materials and methods

### Design and context

In the current cross-sectional study, data was derived from the Youth Health Development (YHD) project, a 3-wave (2010, 2011 and 2012) longitudinal study of health development in adolescents in the northern part of Sweden. The YHD project is part of a governmental investment involving 6 municipalities throughout Sweden. The main purpose of the YHD project was to find methods to promote mental health in adolescents, including development and implementation of such methods in school settings. For the purposes of the current study, data from the 2011 wave was used.

The longitudinal YHD project was conducted in a medium size (60,000 inhabitants) municipality situated in the northern part of Sweden characterized by a diverse socio-economic base with a focus on tourism and small- and medium-sized enterprises. Compared to Sweden in general, the municipality has fewer inhabitants with migrant backgrounds (21). The municipality is situated close to the Norwegian border, implying a relatively high proportion of immigrants from Tröndelag in Norway.

In Sweden, most children begin their first year at school in the fall term when they reach the age of 7 and attendance is compulsory for all children up to the age of 16 or grade level 9. In general, Swedish is used in schools and is spoken by the majority of children in their home environments. However, children who speak a minority language (Finnish, Yiddish, Meänkieli, Romany, Arli, Kalderash, Chib or Sami) have the right to be taught their native language in addition to Swedish (22). The current study focuses on grade levels 7–8 (aged 14–15), and these grade levels are referred to as *high school*.

### Procedure

In the YHD project, all public and independent high schools were invited to participate. All public high schools (n=9) and 1 of 4 independent schools participated. Survey data were collected in January 2011, and information about participation in the DISA program during 2011 was retrieved from the January 2012 wave survey. DISA had been implemented in schools in the geographical area of the YHD project both prior to the start of, and during the project.

As a part of the YHD project methodology, an electronic questionnaire about the psychosocial school environment was administered via student emails during school hours with a research assistant present in the classroom. The questionnaire was in Swedish only and was not translated to any minority language. Informed consent was retrieved from students and parents by written information sent home with the students. Parents and students had to actively decline participation. Students had a second opportunity to decline when receiving the electronic questionnaire by clicking on a link included in the email and by so doing also avoiding a reminder. Students were informed about the aims of the questionnaire and that they could withdraw from participation at any time. The study was approved by the Umeå Regional Ethical Review Board (Dnr 09-179M).

### Participants

The response rate in 2011 of the total sample of the YHD project in grades 7–9 was 80.5 and 79.5% in 2012. For the purposes of the analyses in the current study, girls in grade levels 7 and 8 in 2011 were included (n=288). The non-respondents differed between the schools by 10–40%. Based on this sample size, a post hoc power calculation shows that the current study has a power of 89.2% to detect differences in median scoring of depressive symptoms.

### Measures

Depressive symptoms were measured using the Center for Epidemiological Studies Depression Scale (CES-D) developed for screening rather than for diagnostic purposes (23–25) (scale range 0–60 with higher scores indicating higher levels of depressive symptoms, Cronbach's α=0.89).

Information about the psychosocial school environment was indicated by several variables. *Teacher support* was measured by asking respondents to indicate how often teachers: gave support and help when needed; noticed if they didn't get on with school; treated them fairly; and gave praise or encouragement (always, often, sometimes, rarely, never, scale range 0–16, higher scores indicating more teacher support, Cronbach's α=0.83). An index of *peer support* was constituted by asking how often: Do you have as many friends you'd like?; Are you are alone when you don't want to be?; Do you feel left out by your peer group? (always, often, sometimes, rarely, never, scale range 0–12, higher scores indicating more peer support, Cronbach's α=0.70). *School demands* was measured by 3 items: how often school work moves forward too quickly; what we are supposed to learn is too difficult; and the teachers give us too much homework (26) (always, often, sometimes, rarely, never, scale range 0–16, higher scores indicating more school demands, Cronbach's α=0.73). Exposure to *sexual harassment* was measured using 14 items (27) asking respondents to indicate how often they had experienced each behaviour against their will during the past 6 months. Example of items were: touched, grabbed or pinched you in a sexual manner; spread sexual rumours about you (never, once, a few times, many times, scale range 0–56, higher scores indicating more victimization, Cronbach's α=0.86). *Bullying* was measured by asking respondents to indicate if in the past 6 months it had happened that one or several students have teased, picked fights or shut the respondent out (no, never; yes, once; yes, a few times; yes, several times; yes, almost all the time, scale range 1–5, higher scores indicating higher levels of victimization).

Two dichotomous proxies for socio-economic position of the respondents were used: *Parental employment status* (2 employed parents vs. 1 or 2 unemployed parents); and *personal relative affluence*, that is, respondents were asked to indicate how often in the past 6 months they had had as much money as their friends in order to do the same things as them (always, often, sometimes, rarely, never, collapsed into 2 categories; always/often=yes; sometimes/rarely/never=no).

In the longitudinal YHD study, no items on race or ethnicity of the students were included. *Family migrant background* was measured by asking respondents in what country their mother and father were born. Students with one or both parents from a non-Swedish background were contrasted with students with both parents from Sweden.

Socio-economic status of each school is described using data from The Swedish National Agency for Education (28), which provides data on *proportion of parents with low education* (no more than high school), *proportion of parents with foreign background* and *proportion of students who in 2011 graduated with a high school diploma*.

The proportion of missing data was as follows: CES-D 14.6%; teacher support 7.9%; peer support 4.7%; school demands 2.2%; sexual harassment 5.4%; bullying 3.5%; parental unemployment 6.6%; family migrant background 2.2%; and personal relative affluence 0.9%. No information on missing data regarding the socio-economic status of each school was available (28). Patterns of missing items were calculated using Little’s Missing Completely At Random test (MCAR test) (29). Missing items on sexual harassment and bullying were judged to be not missing at random (NMAR), χ^2^=289.280, DF(97), p<0.001, with a weak negative (−0.154) correlation (p=0.003). Multiple imputations have been conducted to address these issues.

### Statistical analyses

DISA-participating students were classified as intervention participants and all other students were assigned toa comparison group. Because of the skewed distribution of the CES-D as well of all other variables, and because all variables were on the ordinal level, between-group differences in median scoring were analysed using the Mann–Whitney U-test (30). Differences between categorical variables were analysed using chi-squared statistics. The theta (θ) statistics were used to calculate effect sizes of the U statistics by dividing the U by the product of the two sample sizes *mn* (31). Given the positively skewed distributions in both groups, confidence intervals of the effect sizes were calculated using a generalized model (32), which represents a modification of the model used by Hanley and McNeil (33). The significance levels were set to p<0.05. The SPSS PASW Statistics 22 software was used for all statistical analyses.

## Results

In total 66 girls and no boys reported DISA participation during 2011. Because only girls participated in DISA during 2011, girls only (n=222) were assigned to the comparison group. All schools implemented at least 1 DISA group during the same period. The DISA participants clustered mainly at 5 of the 10 schools (i.e.≥10% of the participants at each school, not reported in table). The comparison group clustered mainly at 4 schools. Six (9%) of the girls reported low fidelity (participating on a few occasions). Fifty-three girls participated in the spring term and 13 girls in the fall. Thirty-three (48.5%) girls were in grade 7 (14 years old), while 32 (51.5%) were in grade 8 (15 years old) when participating in DISA (not reported in table).

As shown in Table I, at baseline, DISA participants had a significantly higher median score of depressive symptoms than the comparison group, with a low effect size. DISA participants reported more sexual harassment victimization and less peer support, both with medium effect sizes. There was also a tendency towards a between-group difference in exposure to bullying.

**Table I:** Between-group differences in median scorings of female DISA participants and female non-participants using the Mann–Whitney U statistics

	DISA participants		Non-participants					
						
Variable	n	Median	n	Median	U	z	ES (95% CI)	p
Depressive symptoms	66	16	222	12	5,302	−3.065	0.36 (0.29, 0.44)	0.003
Peer support	62	9	211	10	5,454	−2.013	0.42 (0.34, 0.50)	0.044
Teacher support	65	9	203	10	5,835	−0.686	0.47 (0.39, 0.55)	0.493
School demands	65	8	216	9	6,906	−0.200	0.49 (0.41, 0.57)	0.842
Sexual harassment	65	2	206	0	5,383	−2.259	0.40 (0.33, 0.48)	0.011
Bullying	65	1	212	1	5,933	−1.964	0.43 (0.35, 0.55)	0.050

There were no statistically significant differences in family migrant background or parental unemployment status between the DISA intervention group and the comparison group (Table II). However, relative to the comparison group, a greater proportion of DISA participants had low personal relative affluence (Table II).

**Table II:** Between-group differences in socio-economic variables using the chi-squared statistics

	DISA participants		Non-participants				
					
Variables	n	%	n	%	p	χ^2^	DF
Parental foreign background	7	10.8	43	19.9	0.091	2.852	1
Parental unemployment	10	16.7	18	8.7	0.074	3.196	1
Low personal relative affluence	22	33.3	37	16.8	0.004	8.457	1

In Table III, data from The Swedish National Agency for Education shows that parental educational level as well as the proportion of students receiving a high school diploma was somewhat lower in the schools where DISA participants clustered. However, family migrant background was 6% in both DISA schools and comparison group schools. Note, however, that there are no p-values available for these data.

**Table III:** Description of schools’ socio-economic characteristics

	DISA schools	Comparison schools
	
Variable	%	%
Graduating with a diploma in Year 9	67.3	72.0
Family migrant background	6.0	6.0
	Mean	Mean
Parents with low education^a^ (range 0–3)	2.2	2.4

Data from the SALSA database.

No p-values available for these data. Statistical tests for between-group differences were not possible to perform. Hence, no p-values are presented.

a Mean of each student's birth or adoptive parents’ level of education. Parental education is awarded 1 point if high school (year 9) is completed; 2 points if senior high school (year 12) is completed; and 3 points if an additional fourth year of senior high school or a minimum of 30 ETC in college or university is completed. Parents’ weighted level of education is the mean value of each school.

## Discussion

Our results show that the DISA intervention participants reported higher levels of depressive symptoms than the comparison group. This indicates that DISA was implemented as a targeted intervention for girls with elevated symptoms rather than to girls in general as a selected intervention or to both sexes as a universal intervention as recommended by the developers (11). One possible interpretation is that school staff identified girls with adverse mental health and offered participation in DISA as a way to help these girls. However, when a school-based intervention is implemented as targeted, there is a possibility that students allocated to the intervention are stigmatized which surely is not the intention of school staff. However, no information on students’ satisfaction or general experience of participating was included in the survey. Yet, previous research (34) has shown that the method itself could contribute to stigmatization – participating girls point out that the program presumes a specific problem profile of the participant: a participant who identifies with having low self-esteem, feeling depressed, and having compulsive negative thoughts and in addition being harmed by them. The same authors (34) point out that there is an inherent contradiction in the theoretical underpinnings of the DISA program. In the DISA manual, the methods are defined as preventive (11). At the same time, the theoretical framework of the manual refers to the “field of therapy” and “already depressed adolescents” (11), which highlights the clinical origin of the program (34). Possibly, these writings may be misleading for schools when planning their implementation and may help explain the results in the current study. But, as shown by a previous study (10), DISA did not have an effect on girls with elevated depressive symptoms and should not be implemented by targeting this group of girls.

Implementation participants also reported more sexual harassment, less peer support and lower personal relative affluence compared to non-participants. The schools in which DISA was implemented were more socially burdened relative to the schools in which most of the non-DISA participants were enrolled (28). In the DISA-evaluation study by Garmy et al. (12), the school from which the study population was derived was located in an area with high mean income. This could explain the differences in BL depressive scoring between their study and ours, because our population mirrors a greater socioeconomic diversity.

Previous research (7) shows discrepancies in intervention effects on depressive symptoms depending on schools. For example, in 2 schools the Penn Resiliency Program was superior to another intervention (Penn Enhancement Program) as well as a control condition. In a third school, however, the Penn Resiliency Program was not significantly better than the control condition and was outnumbered by the Penn Enhancement Program (7). The authors were not able to determine the cause of this; however, based on our results, it would be interesting to investigate whether school-specific psychosocial environmental and socio-economic factors underlie these discrepancies.

These results have methodological implications regarding the evaluation of cognitive-behavioural interventions in schools. If participants who are allocated to take part in cognitive-behavioural programs have other characteristics than the program has intended them to have, for example, elevated depressive symptoms and exposure to harassment in addition to an unfavourable socio-economic position, it seems reasonable to assume that effects of the intervention may be different than expected. To explore this further, both naturalistic and randomized controlled designs evaluations should be used in future research. Given the results of the current study, and despite considerable methodological challenges, it is important not to refrain from evaluating effects of interventions as implemented solely by schools as that would likely bias the evidence body of the effects of such methods (17,35).

### Methodological considerations

One strength of the current study is the high response rate. However, all data is based on self-reports, and memory bias may be present because we identified DISA participants in a questionnaire rather than in school records. It may have been difficult to recall the year of participation as the DISA program had been running for several years in at least some of the schools. We utilized data from The Swedish National Agency for Education (28) for information on socio-economic factors on school level. Unfortunately, it was not possible to conduct significance testing on this data because sufficient information was not available. The results regarding school-level socio-economic differences should therefore be interpreted with caution. The internal consistency of all measured variables was acceptable (Cronbach α≥0.70). By using the Mann–Whitney U-test as an alternative for ordinal level data and positively skewed data, as well as data with different sample sizes, we have used the soundest statistic available for this kind of data (30–32). Besides measures of statistical significance, effect size is also reported along with confidence intervals of the effect sizes and shows the degree of separation of the distributions of the implementation and comparison groups.

## Conclusions and implications

The DISA program seems to have been implemented as a targeted intervention for girls with elevated symptoms rather than to girls in general as a selected intervention, or to both sexes as a universal intervention as recommended by the developers (11).

When evaluating effects of cognitive-behavioural interventions that in themselves are only aimed at strengthening individual resilience, the impact of psychosocial environmental factors, for example, sexual harassment and socio-economic factors on individual as well as at school levels, should be acknowledged. This approach could help deepen the understanding of discrepant results in effect trials. As the method to our knowledge so far also has spread to Finland (15), it is important for school administrations in the Nordic countries to adhere to program fidelity when it comes to what students it is aimed for. However, given that schools in this study seem to implement DISA as a targeted intervention and not as a selective one, future studies should investigate whether DISA has an effect when used as a targeted intervention. That said, the effects of the DISA program should also be further evaluated in randomized controlled trials in a Swedish setting in order to draw conclusions of its effects as a selective (or universal) intervention.
